# Uncovering the Role of *ALDH1A2* in Prostate Cancer: Insights from Genetic and Expression Analyses

**DOI:** 10.7150/jca.104705

**Published:** 2025-01-01

**Authors:** Chi-Fen Chang, Bo-Ying Bao, Te-Ling Lu, Lih-Chyang Chen, Yei-Tsung Chen, Tzu-Ping Lin

**Affiliations:** 1 Department of Anatomy, School of Medicine, China Medical University, Taichung 406, Taiwan.; 2 Department of Pharmacy, China Medical University, Taichung 406, Taiwan.; 3 Department of Medicine, Mackay Medical College, New Taipei City 252, Taiwan.; 4 Department of Life Sciences and Institute of Genome Sciences, National Yang Ming Chiao Tung University, Taipei 112, Taiwan.; 5 Department of Urology, College of Medicine and Shu-Tien Urological Research Center, National Yang Ming Chiao Tung University, Taipei 112, Taiwan.; 6 Department of Urology, Taipei Veterans General Hospital, Taipei 112, Taiwan.

**Keywords:** Prostate cancer, recurrence, aldehyde dehydrogenase, gene set enrichment analysis, prognosis

## Abstract

Biochemical recurrence (BCR) is a critical concern in prostate cancer management; however, its underlying genetic determinants remain poorly understood. The *aldehyde dehydrogenase 1* (*ALDH1*) gene family is involved in cellular detoxification and biosynthetic processes and has been implicated in various cancers. This study investigated the association between the *ALDH1* family members and prostate cancer recurrence. We conducted a two-stage genetic association study involving 134 single-nucleotide polymorphisms within the *ALDH1* family to assess their association with BCR-free survival in prostate cancer. Gene set and pathway enrichment analyses were performed to explore the biological relevance of significant genes across multiple datasets. *ALDH1A2* rs16939929 showed a robust association with BCR-free survival in both discovery and replication cohorts. Functional analyses indicated that rs16939929 affected *ALDH1A2* expression in various tissues. Pooled analysis of 42 prostate cancer gene expression datasets revealed that *ALDH1A2* expression was significantly lower in prostate cancer tissues and higher expression was associated with better patient prognosis. Enrichment analyses revealed that *ALDH1A2* was co-expressed with genes primarily involved in cell adhesion pathways. Further analysis confirmed that several of these co-expressed cell adhesion molecules were associated with improved patient survival. In addition, *ALDH1A2* expression was associated with increased immune cell infiltration into the prostate cancer microenvironment. In conclusion, *ALDH1A2* rs16939929 is a significant predictor of BCR-free survival in prostate cancer, potentially through its effects on the gene expressions of *ALDH1A2* and cell adhesion molecules. These findings suggest that *ALDH1A2* plays a tumor-suppressive role in prostate cancer progression.

## Introduction

Prostate cancer is among the most prevalent malignancies in men, ranking as the second most commonly diagnosed cancer worldwide and one of the leading causes of cancer-related mortality 1. Clinical presentations of prostate cancer vary widely, ranging from low-risk localized disease, which can be managed with active surveillance, to high-risk metastatic disease, which leads to significant mortality. Standard treatments such as radical prostatectomy (RP) are often employed for clinically localized prostate cancer; however, approximately one-third of patients experience biochemical recurrence (BCR), which is characterized by rising levels of prostate-specific antigen (PSA) following RP 2. Despite the widespread use of risk stratification systems based on PSA level, Gleason score, and tumor stage, a substantial proportion of patients, particularly those with intermediate-risk profiles, experience unpredictable outcomes, underscoring the need for more precise prognostic tools 3. Genetic factors and tumor heterogeneity further complicate the prognosis and treatment of prostate cancer, as highlighted by genome-wide association studies that have identified numerous susceptibility loci linked to the disease 4, 5. Therefore, the challenges with predicting long-term outcomes persist, necessitating the identification of novel biomarkers and approaches to guide personalized treatment strategies.

The aldehyde dehydrogenase 1 (ALDH1) family belongs to the broader ALDH superfamily, a group of enzymes that facilitate the oxidation of aldehydes to carboxylic acids using nicotinamide adenine dinucleotide phosphate^+^ as a coenzyme 6. This enzymatic activity is crucial for detoxifying aldehydes generated by various metabolic processes, thereby protecting cells from damage. ALDH1 plays key roles in the biosynthesis of retinoic acid (RA), a vital regulator of gene expression during development and cellular differentiation 7. The *ALDH1* family performs a complex role in cancer biology, with functions ranging from promoting tumor growth to tumor suppression. Specific *ALDH1* isoforms, such as *ALDH1A1* and *ALDH1A3*, are highly expressed in certain cancer cell subpopulations that exhibit stem cell-like characteristics and are resistant to chemotherapy and radiotherapy. *ALDH1*-positive cancer stem-like cells are thought to drive tumor initiation, progression, and recurrence 8. *ALDH1A1* has been shown to influence the immune microenvironment in breast cancer by promoting the expansion of myeloid-derived suppressor cells that facilitate tumor progression 9. Targeting *ALDH1A3* with a selective inhibitor and short hairpin RNA has the potential to reduce cancer cell stemness, migration, and invasiveness, making it a promising approach in cancer therapy 10. Conversely, the *ALDH1* isoform *ALDH1A2* has been suggested to act as a tumor suppressor in ovarian cancer, where its expression is significantly reduced, correlating with poor patient prognosis 11. Recent studies have also investigated the association between genetic variants of *ALDH1* family members and cancer susceptibility. For instance, *ALDH1A1* rs1330286 and *ALDH1A3* rs4646653 have been linked to the risk of prostate cancer 12, while *ALDH1L1* rs2276724 has been associated with prognostic outcomes in patients with hepatocellular carcinoma 13. Additionally, several single-nucleotide polymorphisms (SNPs) in *ALDH1A1* have been linked to an increased risk of breast cancer mortality 14. However, the clinical relevance of these genetic variants in prostate cancer progression remains unclear.

Given the pivotal roles of *ALDH1* family in cellular detoxification and biosynthetic regulation, we hypothesized that variants of these genes could influence prostate cancer outcomes. To test this hypothesis, a two-stage genetic association study was conducted that systematically evaluated 134 SNPs in these genes among 457 patients with prostate cancer who experienced BCR and then validated our findings in an independent cohort of 187 patients. Subsequent functional analyses, including gene ontology and pathway enrichment analyses, provided insights into the biological mechanisms by which *ALDH1A2* influences prostate cancer progression.

## Patients and Methods

### Study population and data collection

This study recruited 644 patients with histopathologically confirmed prostate cancer who had undergone RP at three medical centers in Taiwan: Kaohsiung Medical University Hospital, Kaohsiung Veterans General Hospital, and National Taiwan University Hospital, as previously reported 15, 16. All participants were of Han Taiwanese ethnicity, unrelated, and had not received adjuvant hormone therapy or radiotherapy after RP. Data on demographic and clinical variables such as age at diagnosis, PSA levels, pathologic Gleason score, cancer stage, surgical margin status, lymph node metastasis, and BCR status were obtained from medical records. BCR was defined as two successive PSA readings of 0.2 ng/mL or higher following RP 17, 18. The BCR-free survival time was calculated from the date of RP to either the occurrence of BCR or last follow-up. The study protocol was reviewed and approved by the Institutional Review Board of Kaohsiung Medical University Hospital (KMUHIRB-2013132). Written informed consent was obtained from all participants in accordance with the institutional requirements.

To assess the influence of genetic variants within *ALDH1* family genes on patient prognosis, a two-stage approach was employed. The initial discovery cohort included 457 participants recruited from National Taiwan University Hospital and E-Da Hospital. For validation, a replication cohort of 187 individuals was assembled from Kaohsiung Medical University Hospital. The baseline clinical and pathological characteristics of the discovery and replication sets are summarized in Table S1, which shows no significant differences in variables such as age at diagnosis, PSA levels, pathologic Gleason score, and lymph node metastasis between the two groups. Variables such as PSA level at diagnosis, pathological Gleason score, and stage were significantly associated with BCR-free survival in both groups. The median follow-up periods were 38 and 74 months for the discovery and replication sets during which 137 (30.0%) and 92 (49.2%) patients, respectively, experienced BCR.

### SNP selection and genotyping

Haplotype tagging SNPs were selected across six genes of the *ALDH1* family, *ALDH1A1*, *ALDH1A2*, *ALDH1A3*, *ALDH1B1*, *ALDH1L1*, and *ALDH1L2*, using Haploview v4.2 software, with criteria including a minor allele frequency (MAF) greater than 0.05 and a pairwise linkage disequilibrium correlation coefficient (r²) exceeding 0.8, based on data from the 1000 Genomes Project, specifically for Han Chinese individuals in Beijing and Southern Han Chinese populations 19, 20. Genomic DNA was extracted from peripheral blood lymphocytes using the QIAamp DNA Blood Maxi Kit (Qiagen, Valencia, CA, USA), according to the manufacturer's protocol. Genotyping was carried out at the National Center for Genome Medicine in Taiwan using the Affymetrix Axiom Genotyping Array system (Thermo Fisher Scientific, Waltham, MA, USA), following established procedures 21. SNPs that did not meet the quality control thresholds, a genotyping call rate of at least 95%, a MAF of 0.05 or higher, and compliance with Hardy-Weinberg equilibrium (*P*>0.001), were excluded from further analysis. As a result, 134 SNPs were ultimately retained for subsequent investigation.

### Bioinformatic analyses

We performed expression and splicing quantitative trait loci analyses using the FIVEx database to investigate the genetic influences on *ALDH1A2* expression 22. Functional predictions for rs16939929 were conducted using HaploReg v4.2. 23. To assess the relationship between *ALDH1A2* expression and clinical outcomes in prostate cancer, we analyzed 42 publicly available datasets from PCaDB 24, the Gene Expression Database of Normal and Tumor Tissues 2 25, and The Cancer Genome Atlas (TCGA). The low- and high-expression groups were stratified based on median expression values. We further explored the molecular mechanisms linked to *ALDH1A2* by examining gene correlations in TCGA Prostate Adenocarcinoma (PRAD) samples using Pearson's correlation through LinkedOmics 26, followed by gene ontology terms and Kyoto Encyclopedia of Genes and Genomes (KEGG) pathway enrichment analyses using gene set enrichment analysis (GSEA) with a false discovery rate (FDR) threshold of <0.05, and 1,000 permutations. The prognostic significance of *ALDH1A2*, along with cell adhesion molecule genes such as junctional adhesion molecule 3 (*JAM3*), neural cell adhesion molecule 1 (*NCAM1*), and neuronal growth regulator 1 (*NEGR1*), were evaluated using the TCGA-PRAD dataset, and the infiltration levels of tumor-infiltrating immune cells were analyzed in relation to *ALDH1A2* gene alterations and expression using the tumor immune estimation resource (TIMER) 27.

### Statistical analyses

Survival differences across genotypes or gene expression groups were evaluated using Kaplan-Meier analysis and log-rank tests, whereas univariate and multivariate Cox regression analyses were used to determine the associations between clinicopathological features and patient prognosis by calculating hazard ratios (HRs) with 95% confidence intervals (CIs). The correlation between *ALDH1A2* expression and tumor characteristics was assessed using Pearson's and Spearman's correlations. Statistical analyses were performed using the SPSS software v19.0.0 (IBM, Armonk, NY, USA), with a two-sided *P*-value of less than 0.05 considered statistically significant. Additionally, *ALDH1A2* expression in prostate cancer and adjacent normal tissues were compared using standardized mean differences (SMD) with 95% CIs, and the association between *ALDH1A2* mRNA expression and survival outcomes was analyzed by pooling HRs and CIs via a random-effects model using Review Manager v5.4.1 (Cochrane, London, UK).

## Results

**Figure 1:**
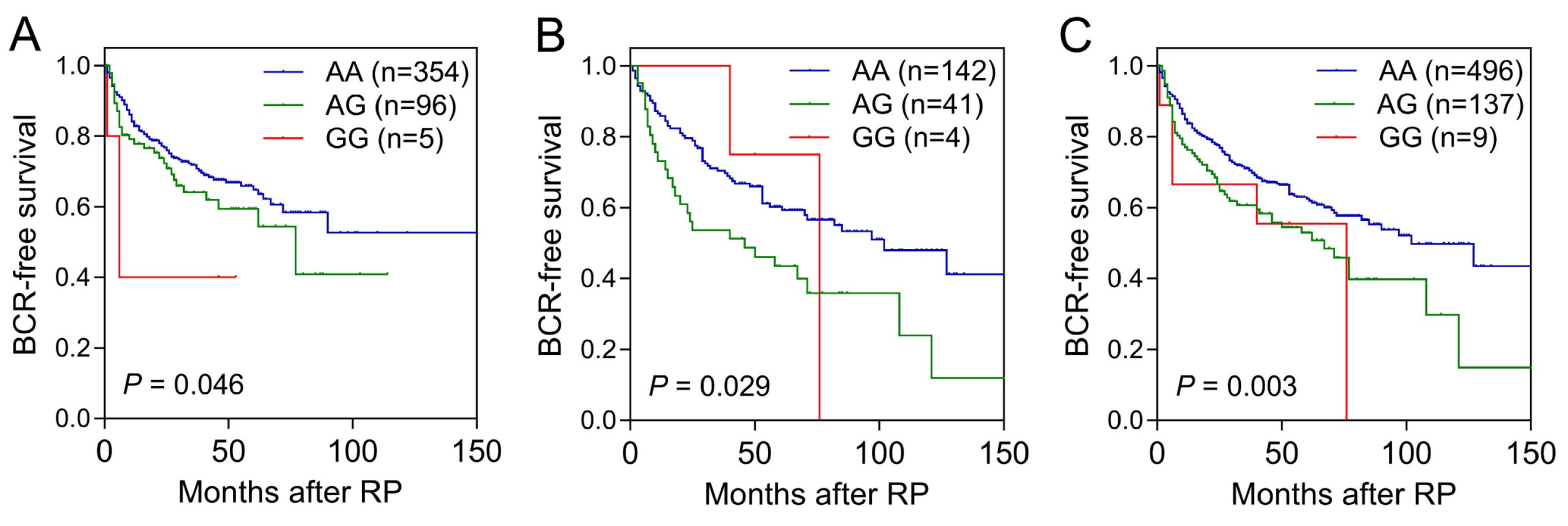
Kaplan-Meier survival curves illustrating biochemical recurrence (BCR)-free survival according to *ALDH1A2* rs16939929 genotype in the (A) discovery, (B) replication, and (C) combined cohorts. Log-rank *P*-values for each analysis are indicated. RP: radical prostatectomy.

Cox regression analysis was used to examine the relationship between *ALDH1* family genetic variants and risk of BCR (Table S2). In the discovery set, out of the 134 SNPs analyzed, five SNPs within *ALDH1A2* and one SNP within *ALDH1L2* were significantly associated with BCR-free survival (*P*<0.05). Notably, an association with *ALDH1A2* rs16939929 was consistently observed in the replication cohort (*P*=0.031; Table 1 and Figure 1B). When combined, the *ALDH1A2* rs16939929 A>G variant showed a significant adverse effect on BCR-free survival. This association remained robust in multivariate Cox analyses, which accounted for clinical risk factors such as age, PSA levels at diagnosis, pathological stage, Gleason score, surgical margin status, and lymph node metastasis (adjusted HR=1.49, 95% CI=1.12‒1.98, *P*=0.007; Table 1 and Figure 1C).

**Figure 2:**
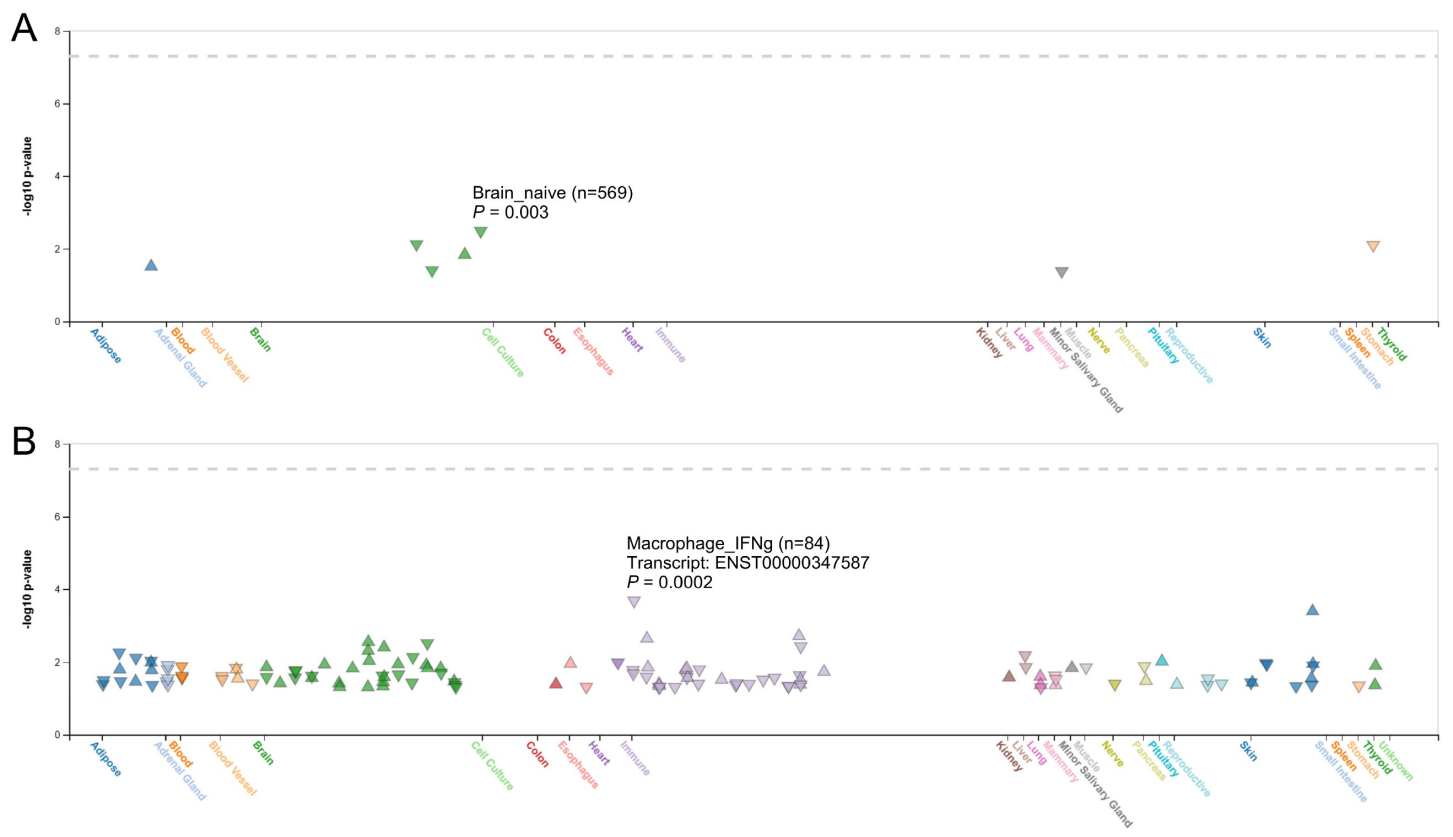
Correlation between rs16939929 genotypes and *ALDH1A2* expression in various tissues. (A) Analysis of expression quantitative trait loci, and (B) analysis of splicing quantitative trait loci. Triangles indicate a positive effect of rs16939929 on *ALDH1A2* expression, while inverted triangles indicate a negative effect. To refine the results, filters were applied with x-axis grouping by system, ±1 Mb from the variant to the nearest gene, -log_10_ *P* > 1.3, and gene match to *ALDH1A2*.

To further explore the functional relevance of rs16939929, we used the HaploReg database, which suggested that this SNP may play a regulatory role because of its location within enhancer histone marks across multiple tissues and its effect on transcription factor-binding motifs (Table S3). The FIVEx database supported these findings, showing that the risk G allele was associated with reduced *ALDH1A2* expression in the brain, salivary glands, and stomach (Figure 2A). Additionally, splicing quantitative trait loci analysis revealed significant associations between rs16939929 and various *ALDH1A2* transcripts in multiple human tissues (Figure 2B).

**Figure 3:**
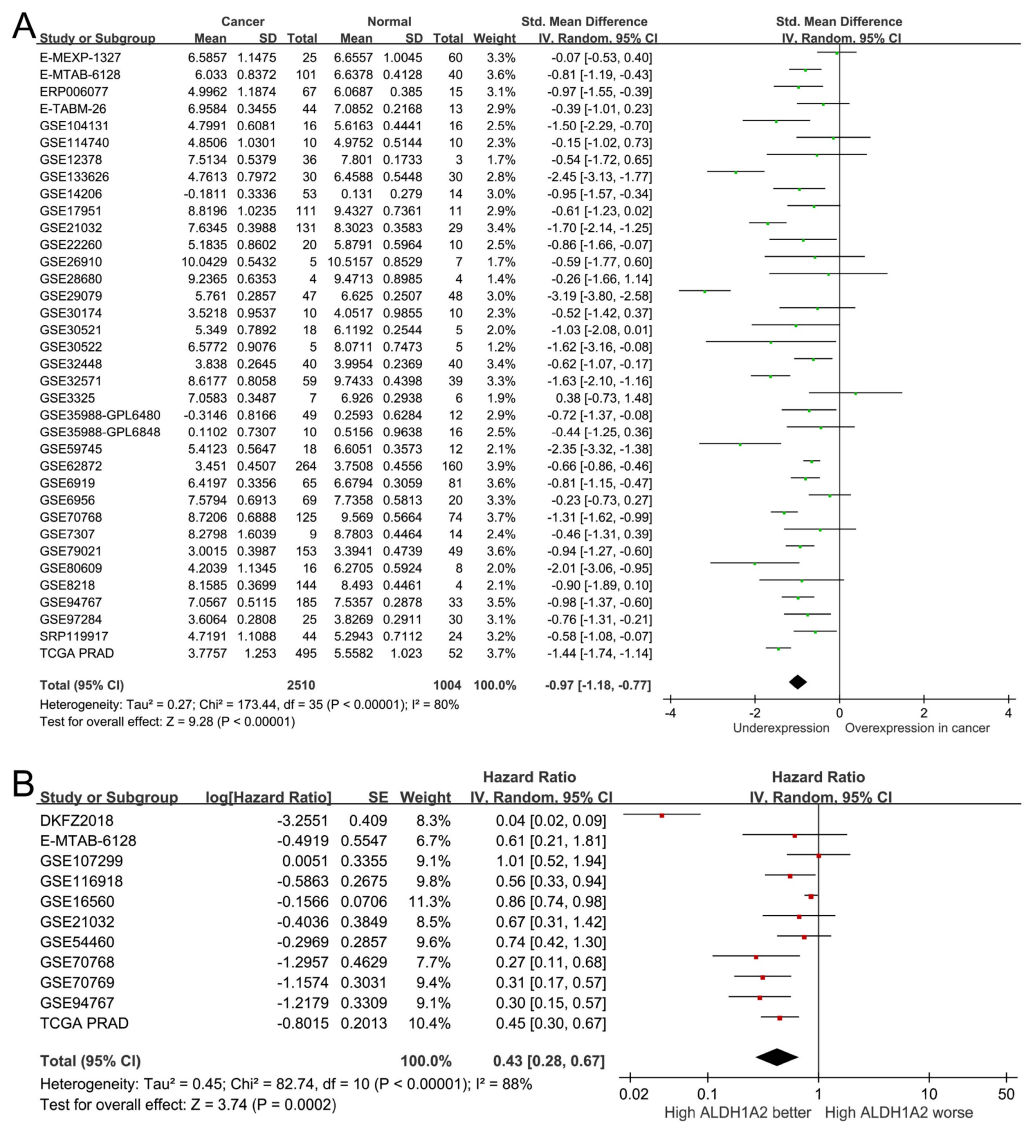
*ALDH1A2* expression levels in prostate cancer. (A) Forest plot showing differential expression of *ALDH1A2* between cancerous and normal prostate tissues. (B) Forest plot showing the relationship between *ALDH1A2* expression and prostate cancer prognosis. SD: standard deviation. SE: standard error. IV: inverse variance. CI: confidence interval. Std: standardized. TCGA PRAD: The Cancer Genome Atlas Prostate Adenocarcinoma. df: degrees of freedom.

To assess the clinical significance of *ALDH1A2* expression in patients with prostate cancer, we analyzed 2,510 prostate cancer samples and 1,004 normal prostate specimens from 36 public datasets. *ALDH1A2* expression was significantly lower in prostate cancer tissues compared to normal tissues (SMD=-0.97, 95% CI=-1.18 to -0.77, *P*<0.001; Figure 3A). A pooled analysis of 11 studies revealed that higher *ALDH1A2* expression was associated with better prognosis (HR=0.43, 95% CI=0.28-0.67, *P*<0.001; Figure 3B), indicating that *ALDH1A2* may play a tumor-suppressive role in prostate cancer progression.

**Figure 4:**
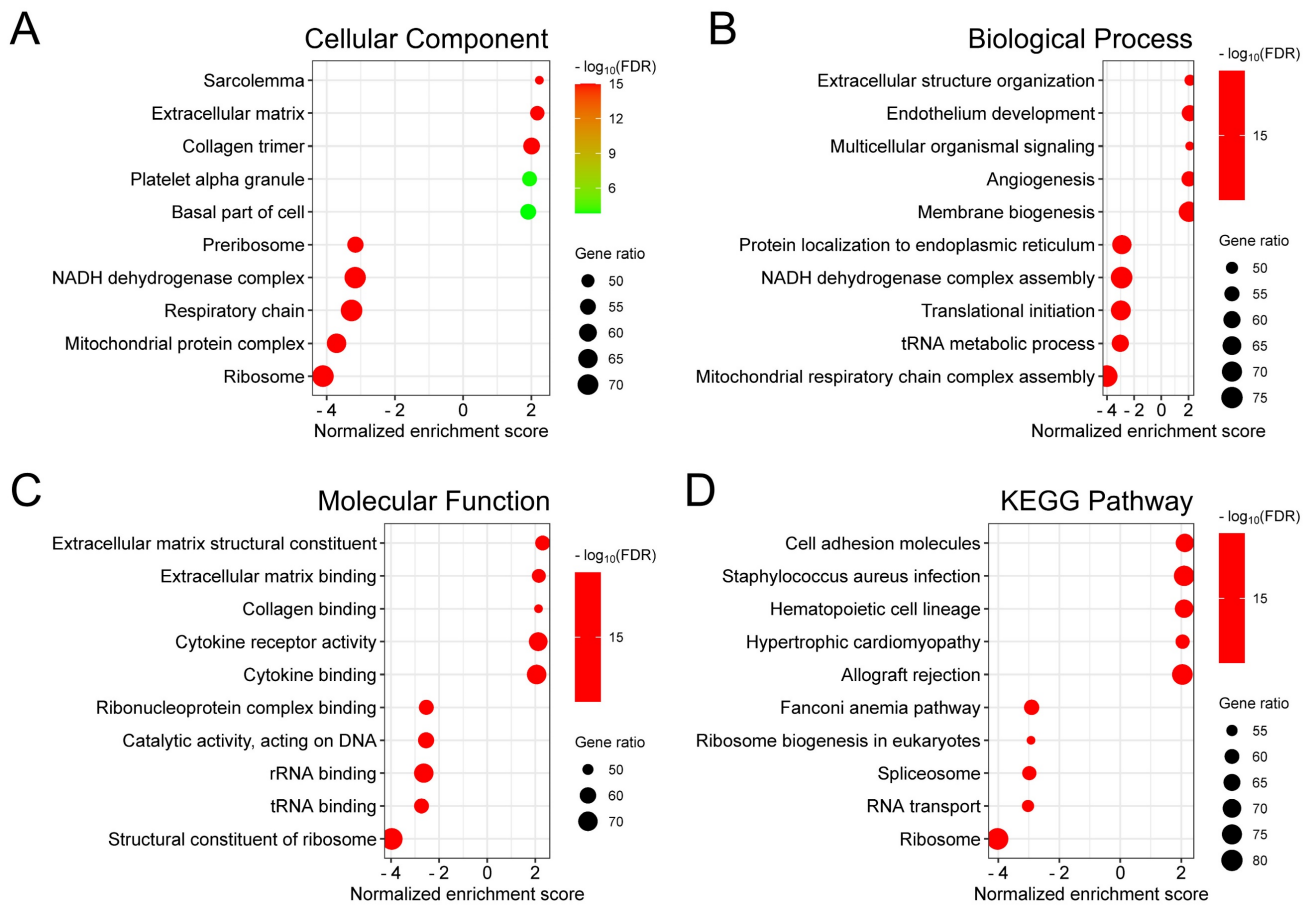
Biological functions of *ALDH1A2* in prostate cancer. Gene ontology annotations for (A) cellular components, (B) biological processes, and (C) molecular functions, and (D) Kyoto Encyclopedia of Genes and Genomes (KEGG) pathway enrichment analysis of genes associated with *ALDH1A2*.

To investigate the biological significance of *ALDH1A2* in prostate cancer, we identified genes that correlated with *ALDH1A2* expression in the TCGA-PRAD dataset. We found 7,019 genes positively and 4,349 genes negatively correlated with *ALDH1A2*, with an FDR of less than 0.01, based on Pearson's correlation. Gene Ontology analysis revealed that *ALDH1A2*-correlated genes were involved in cellular components, such as the sarcolemma, extracellular matrix, and collagen trimer (Figure 4A). These genes also participate in biological processes, including extracellular structure organization, endothelium development, and multicellular organismal signaling (Figure 4B), and are associated with molecular functions such as extracellular matrix structural constituents, extracellular matrix binding, and collagen binding (Figure 4C). KEGG pathway enrichment analysis showed that genes correlated with *ALDH1A2* expression were primarily associated with the regulation of cell adhesion molecules and ribosomal translation processes (Figure 4D).

**Figure 5:**
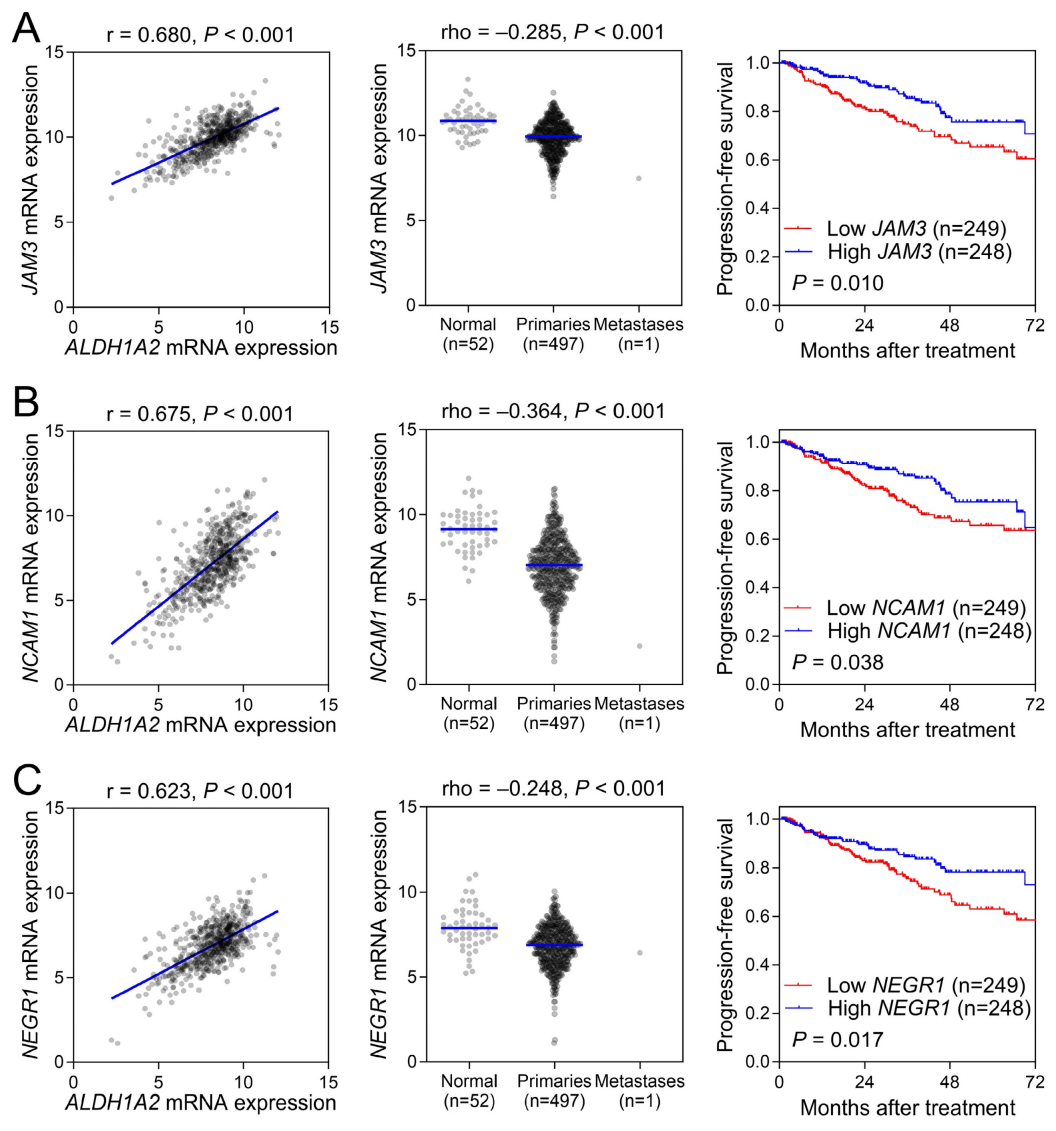
Validation of cell adhesion molecule genes, including (A) *JAM3*, (B) *NCAM1*, and (C) *NEGR1*, in prostate cancer. The left panel shows a positive correlation between *ALDH1A2* and the cell adhesion molecule genes in The Cancer Genome Atlas Prostate Adenocarcinoma dataset. The middle panel shows a lower expression of cell adhesion genes in prostate cancer samples than in adjacent normal tissues. The right panel shows the association between the expression of cell adhesion genes and progression-free survival in patients with prostate cancer.

To validate the role of cell adhesion molecules in prostate cancer, we selected genes involved in cell matrix adhesion that were co-expressed with *ALDH1A2*. Our results showed that *ALDH1A2* positively correlated with the expression of *JAM3*, *NCAM1*, and *NEGR1* (Figure 5, left). A comparison of gene expression levels between prostate cancer and normal tissues in the TCGA-PRAD dataset revealed significantly lower expression of these cell adhesion molecules in prostate cancer (Figure 5, middle). Moreover, the expression levels of these cell adhesion molecules were significantly associated with improved patient survival, further supporting the potential protective role of *ALDH1A2* in prostate cancer progression through these cell adhesion molecules.

**Figure 6:**
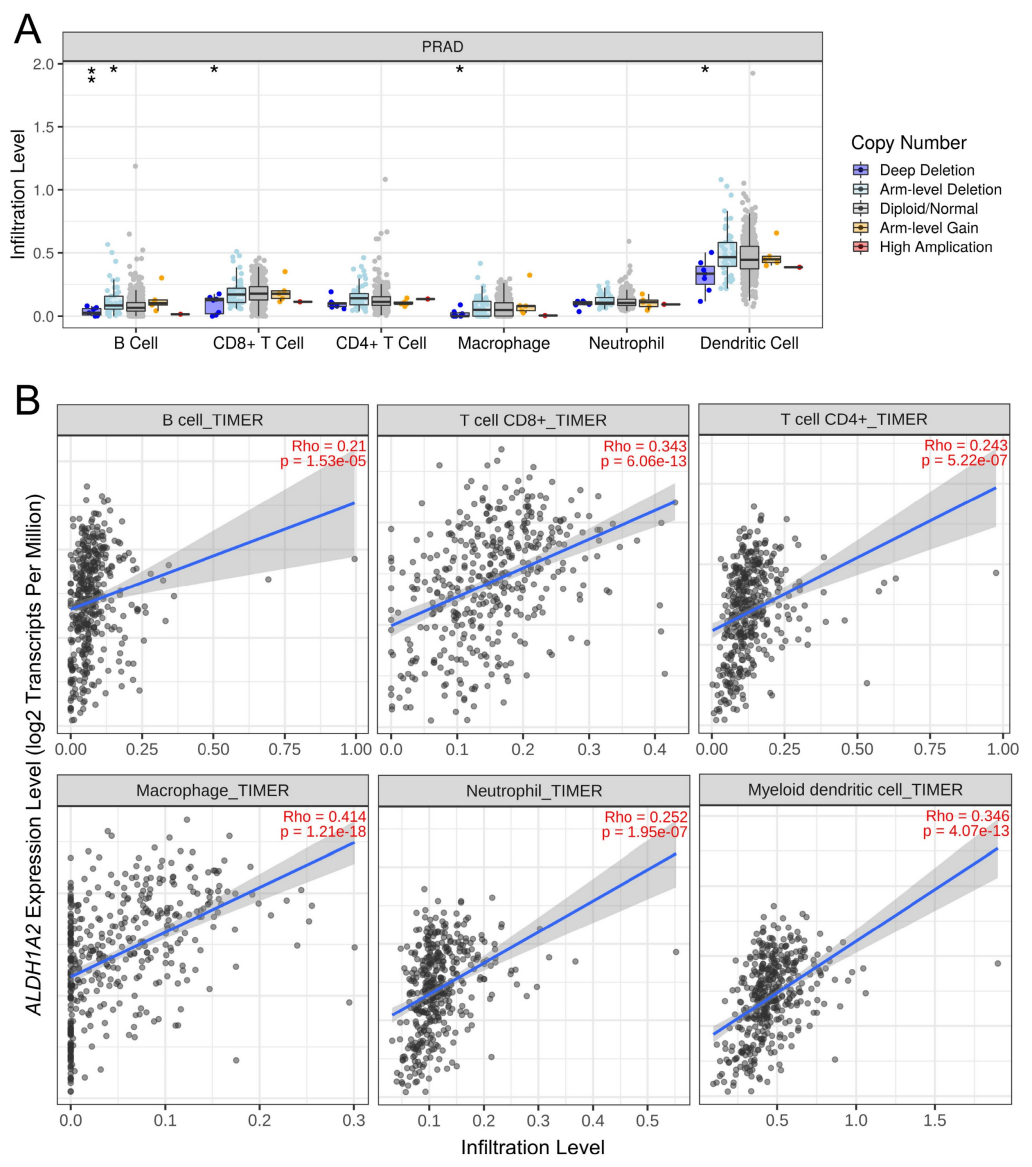
Association between *ALDH1A2* expression and immune cell infiltration in the prostate cancer microenvironment. (A) Impact of *ALDH1A2* copy number variation on the infiltration levels of different immune cell types in The Cancer Genome Atlas Prostate Adenocarcinoma dataset. *P < 0.05; **P < 0.01. (B) Correlation between *ALDH1A2* expression and infiltration of B cells, CD8⁺ T cells, CD4⁺ T cells, macrophages, neutrophils, and dendritic cells.

Given the known association between *ALDH1* family members and the tumor immune microenvironment, we explored the relationship between *ALDH1A2* expression and immune cell infiltration in prostate cancer. Deep deletion of *ALDH1A2* was linked to reduced infiltration of B cells, CD8^+^ T cells, macrophages, and dendritic cells (Figure 6A). Additionally, higher *ALDH1A2* expression was positively correlated with the infiltration of all measured immune cells, including B cells, CD8^+^ T cells, CD4^+^ T cells, macrophages, neutrophils, and dendritic cells, in prostate cancer (Figure 6B). These findings suggested that *ALDH1A2* plays a crucial role in modulating immune cell infiltration in prostate cancer.

## Discussion

In this study, we investigated the relationship between genetic variants of the *ALDH1* family and the risk of BCR in prostate cancer. *ALDH1A2* rs16939929 consistently showed a significant effect on BCR-free survival in both discovery and replication cohorts. Functional analyses indicated that rs16939929 may regulate *ALDH1A2* expression, as the G allele was associated with reduced *ALDH1A2* expression. Additionally, *ALDH1A2* expression was significantly lower in prostate cancer tissues than in normal tissues, and higher *ALDH1A2* expression correlated with better patient prognosis. Gene set and pathway enrichment analyses revealed that *ALDH1A2*-related genes were involved in cell adhesion, and that these co-expressed cell adhesion molecules were also linked to improved survival. Furthermore, higher *ALDH1A2* expression was positively associated with immune cell infiltration, suggesting a protective role in prostate cancer progression.

The SNP rs16939929 is located within an intronic region that displays enhancer-like chromatin modification patterns and influences the binding affinity of multiple transcription factors. This suggests a potential role for rs16939929 in the regulation of *ALDH1A2* expression. Our expression quantitative trait loci analysis indicated that the risk G allele is associated with decreased *ALDH1A2* expression in various human tissues. However, rs16939929 demonstrated only a trend toward correlation with decreased *ALDH1A2* expression in prostate, and was even associated with increased expression in certain other tissues. The variability of genetic variants and their functional impacts across cell types and tissues highlights a cellular context-dependent effect 28. This implies that the influence of rs16939929 on *ALDH1A2* expression and transcription factor binding may vary depending on the specific cellular or tissue environment.

Although the *ALDH1* family plays complex roles in cancer biology, *ALDH1A2* has been shown to be downregulated in several tumor types, including prostate cancer, according to TCGA database. In epithelial ovarian cancer, *ALDH1A2* suppresses signal transducer and activator of transcription 3 (STAT3) signaling, a pathway known to promote tumor proliferation and migration. STAT3 enhances cell adhesion by upregulating molecules such as E-selectin and P-selectin in endothelial cells, thereby facilitating cancer cell invasion and metastasis 29. In cancer immunity, STAT3 activation in tumor-associated immune cells promotes the production of immunosuppressive cytokines, including interleukin (IL)-6 and IL-10, while suppressing antitumor cytokines like IL-12 30. Overexpression of *ALDH1A2* significantly diminishes STAT3 activation, resulting in reduced cell proliferation and migration 31. In prostate cancer, *ALDH1A2* is frequently silenced by promoter hypermethylation, leading to decreased RA synthesis, which is essential for normal cellular differentiation. Restoration of *ALDH1A2* expression and RA treatment have been shown to inhibit prostate cancer cell growth by inducing differentiation and reducing proliferation 32. In this study, we explored the clinical significance and function of *ALDH1A2* expression in prostate cancer using bioinformatic and functional analyses. A pooled analysis of multiple datasets revealed that *ALDH1A2* expression was lower in prostate tumors and that patients with higher *ALDH1A2* expression had longer survival times. GSEA of the *ALDH1A2* co-expression network showed enrichment of cell adhesion molecules, such as *JAM3*, *NCAM1*, and *NEGR1*. These molecules and *ALDH1A2* are implicated in tumor suppression through cell-cell and cell-matrix interactions. *JAM3* exhibits frequent hypermethylation and concomitant downregulation in colorectal cancer. Reduced *JAM3* expression is associated with enhanced tumor cell viability and migration. Re-expression of *JAM3* through demethylation strategies results in the suppression of tumor cell growth and migration 33. Similarly, *NCAM1* is expressed at lower levels in bladder cancer cell lines than in normal urothelial epithelial cells and its silencing enhances bladder cancer cell proliferation 34. *NEGR1*, which is commonly downregulated in various cancers, inhibits the proliferation and anchorage-independent growth of SKOV-3 ovarian cancer cells, and its depletion promotes migration and invasion 35. Moreover, high *ALDH1A2* expression correlates with increased infiltration of anticancer immune cells, suggesting that *ALDH1A2* may exert a tumor-suppressive effect in prostate cancer by modulating cell adhesion and anticancer immunity. Notably, *ALDH1A2* expression was significantly reduced in 17 TCGA tumor types, including bladder, breast, cervical, colon, esophageal, glioblastoma, head and neck, kidney, lung, prostate, rectum, stomach, thyroid, and endometrial cancers, when compared to adjacent normal tissues (data not shown). However, further research is required to fully understand the molecular mechanisms by which *ALDH1A2* influences prostate cancer.

Although this study provides valuable insights, it has several limitations that should be acknowledged. The relatively modest sample size across our discovery and replication cohorts may have constrained our ability to detect significant associations, necessitating a cautious interpretation of the results. Additionally, the Taiwanese participant pool limited the generalizability of our findings to other ethnic groups. The absence of detailed clinical information from public datasets and target tissues in the present study further restricted our analysis and mechanistic understanding, highlighting the need for additional research to explore the molecular mechanisms and validate these findings.

Through a discovery-replication design, our study provides compelling evidence that genetic variants in the *ALDH1* family, particularly *ALDH1A2* rs16939929 A>G, may serve as promising prognostic biomarkers for prostate cancer. Functional studies suggest that this variant may influence prostate cancer progression through its association with *ALDH1A2* expression, which is linked to cell adhesion pathways. The identification of survival-associated loci within *ALDH1A2* suggests a broader role in disease etiology, offering new insights into therapeutic targeting and clinical management. However, further large-scale studies and mechanistic investigations are essential to validate these findings and fully elucidate the underlying mechanisms.

## Supplementary Material

Supplementary tables.

## Figures and Tables

**Table 1:** Association of *ALDH1A2* rs16939929 with biochemical recurrence after radical prostatectomy

Genotype	Discovery				Replication				Combined			
	Patients	BCR	HR (95% CI)	*P*	Patients	BCR	HR (95% CI)	*P*	HR (95% CI)	*P*	HR (95% CI)^a^	*P* ^a^
AA	354	100	1.00		142	63	1.00		1.00		1.00	
AG	96	34	1.33 (0.90-1.96)	0.156	41	27	1.83 (1.16-2.87)	0.009	1.50 (1.12-2.01)	0.007	1.47 (1.05-2.05)	0.026
GG	5	3	2.96 (0.94-9.34)	0.065	4	2	1.13 (0.28-4.64)	0.864	1.82 (0.75-4.43)	0.189	2.34 (0.95-5.81)	0.066
Trend			1.42 (1.00-2.00)	0.048			1.50 (1.04-2.16)	0.031	1.45 (1.13-1.87)	0.004	1.49 (1.12-1.98)	0.007

Abbreviations: BCR: biochemical recurrence; HR: hazard ratio; CI: confidence interval; PSA: prostate-specific antigen. ^a^ Adjustment for age, PSA at diagnosis, pathologic stage, Gleason score, surgical margin, and lymph node metastasis.
