# Supplementary material for: Uncovering the Role of ALDH1A2 in Prostate Cancer: Insights from Genetic and Expression Analyses
Source: J Cancer. 2025 Jan 1;16(3):932–41. doi: 10.7150/jca.104705 (PMC11705053; doi:10.7150/jca.104705)
Supplement: Supplementary file 1 — Supplementary tables. [file jcav16p0932s1.pdf]

Table S1. Clinicopathologic characteristics of the study populations

Characteristic	Discovery (n = 457)	Replication (n = 187)
Age at diagnosis		
Median, years (IQR)	66 (62-70)	66 (61-70)
PSA at diagnosis		
Median, ng/ml (IQR)	10.9 (6.9-18.4)	10.8 (7.1-17.6)
Pathologic stage, n (%)		
T1/T2	235 (51.4)	129 (70.9)
T3/T4/N1	222 (48.6)	53 (29.1)
Pathologic Gleason score, n (%)		
2-7	371 (81.2)	160 (85.6)
8-10	86 (18.8)	27 (14.4)
Surgical margin, n (%)		
Negative	307 (67.2)	153 (81.8)
Positive	150 (32.8)	34 (18.2)
Lymph node metastasis, n (%)		
Negative	325 (94.5)	180 (97.8)
Positive	19 (5.5)	4 (2.2)
Biochemical recurrence, n (%)	137 (30.0)	92 (49.2)
Median follow-up, months	38	74

Abbreviations: IQR: interquartile range; PSA: prostate-specific antigen.

Subtotals do not sum to n of patients due to missing data.

Table S2. Association between aldehyde dehydrogenase 1 family gene polymorphisms and biochemical recurrence after radical prostatectomy

Gene	SNP ID	Chromosome	Position	Allele	MAF	HWE	Discovery	Replication
ALDH1A1	rs8187998	9	72901146	T>C	0.056	0.385	0.360	
ALDH1A1	rs4646547	9	72902666	C>A	0.415	0.564	0.868	
ALDH1A1	rs610529	9	72918408	G>A	0.483	0.399	0.604	
ALDH1A1	rs2288086	9	72925656	G>A	0.079	1.000	0.929	
ALDH1A1	rs13959	9	72930966	G>A	0.465	0.224	0.222	
ALDH1A1	rs348463	9	72932696	C>T	0.473	0.302	0.146	
ALDH1A1	rs76100846	9	72933110	A>G	0.064	1.000	0.503	
ALDH1A1	rs146758119	9	72933119	T>C	0.052	1.000	0.998	
ALDH1A1	rs10869208	9	72993710	T>C	0.482	0.350	0.208	
ALDH1A1	rs6560311	9	72997266	T>G	0.481	0.456	0.208	
ALDH1A1	rs7853400	9	73014044	A>G	0.453	0.300	0.348	
ALDH1A1	rs139321864	9	73018961	G>C	0.067	0.449	0.364	
ALDH1A1	rs4745209	9	73025189	C>T	0.472	0.191	0.502	
ALDH1A1	rs4551429	9	73057029	C>T	0.422	0.566	0.279	
ALDH1A2	rs3204690	15	57954570	T>G	0.308	0.231	0.184	
ALDH1A2	rs3204689	15	57954604	G>C	0.202	0.059	0.442	
ALDH1A2	rs3784262	15	57960908	T>C	0.213	0.070	0.446	
ALDH1A2	rs11071365	15	58041928	G>A	0.203	0.313	0.350	
ALDH1A2	rs2464469	15	58069827	G>A	0.379	1.000	0.586	
ALDH1A2	rs28589660	15	58079131	T>C	0.265	0.811	0.744	
ALDH1A2	rs12916872	15	58083542	T>C	0.165	0.735	0.869	
ALDH1A2	rs2642654	15	58104774	T>C	0.052	0.625	0.089	
ALDH1A2	rs58890367	15	58107107	T>C	0.150	0.582	0.008	0.265

ALDH1A2	rs12906402	15	58134436	A>G	0.365	0.312	0.608	
ALDH1A2	rs55913232	15	58147736	T>C	0.222	0.787	0.864	
ALDH1A2	rs12441254	15	58151625	T>C	0.343	0.039	0.094	
ALDH1A2	rs4544186	15	58153835	C>A	0.091	0.408	0.536	
ALDH1A2	rs16953360	15	58155995	A>G	0.060	0.069	0.281	
ALDH1A2	rs75538080	15	58157558	G>A	0.136	1.000	0.971	
ALDH1A2	rs10444840	15	58158075	G>A	0.226	0.594	0.999	
ALDH1A2	rs16939839	15	58166876	T>C	0.100	0.437	0.348	
ALDH1A2	rs17848103	15	58166918	T>C	0.059	0.390	0.736	
ALDH1A2	rs2292711	15	58172748	T>C	0.096	0.786	0.102	
ALDH1A2	rs3858890	15	58178826	G>A	0.204	0.021	0.232	
ALDH1A2	rs16939881	15	58179780	G>C	0.071	0.487	0.635	
ALDH1A2	rs2899619	15	58180007	T>C	0.428	0.294	0.334	
ALDH1A2	rs17240650	15	58186809	T>C	0.118	0.496	0.083	
ALDH1A2	rs17821135	15	58203771	C>T	0.071	1.000	0.945	
ALDH1A2	rs16939929	15	58204734	A>G	0.118	0.658	0.048	0.031
ALDH1A2	rs2017566	15	58212841	C>T	0.315	0.083	0.279	
ALDH1A2	rs1663252	15	58233682	T>C	0.383	0.693	0.067	
ALDH1A2	rs67026867	15	58239877	C>T	0.337	0.917	0.449	
ALDH1A2	rs34458264	15	58257544	C>T	0.484	0.779	0.269	
ALDH1A2	rs72739122	15	58257753	G>T	0.102	0.206	0.444	
ALDH1A2	rs4471613	15	58259495	G>A	0.062	1.000	0.663	
ALDH1A2	rs28690720	15	58281634	T>G	0.097	0.292	0.457	
ALDH1A2	rs12910051	15	58288326	A>G	0.477	0.709	0.426	
ALDH1A2	rs79735913	15	58292496	G>A	0.056	1.000	0.838	

ALDH1A2	rs1711042	15	58298449	G>A	0.327	0.139	0.140	
ALDH1A2	rs261264	15	58322735	C>T	0.414	0.773	0.862	
ALDH1A2	rs17821220	15	58330908	G>C	0.105	1.000	0.205	
ALDH1A2	rs1444940	15	58332922	T>G	0.059	0.663	0.042	0.619
ALDH1A2	rs75004987	15	58333037	T>C	0.057	1.000	0.046	0.799
ALDH1A2	rs56996176	15	58334591	C>T	0.150	0.854	0.723	
ALDH1A2	rs80138407	15	58342102	G>A	0.062	0.087	0.577	
ALDH1A2	rs4775031	15	58343384	C>A	0.200	0.771	0.434	
ALDH1A2	rs444258	15	58353360	G>A	0.166	0.239	0.461	
ALDH1A2	rs8035357	15	58353522	T>C	0.064	0.708	0.177	
ALDH1A2	rs191793	15	58361464	T>C	0.198	0.769	0.619	
ALDH1A2	rs77942490	15	58369341	T>C	0.064	1.000	0.138	
ALDH1A2	rs28829404	15	58379641	T>C	0.058	0.387	0.496	
ALDH1A2	rs4775041	15	58382496	G>C	0.210	0.889	0.953	
ALDH1A2	rs187775	15	58385696	A>T	0.127	0.291	0.983	
ALDH1A2	rs431701	15	58385774	C>T	0.089	1.000	0.858	
ALDH1A2	rs10468017	15	58386313	C>T	0.196	0.553	0.743	
ALDH1A2	rs261290	15	58386521	C>T	0.320	0.915	0.998	
ALDH1A2	rs261291	15	58387979	T>C	0.424	0.504	0.637	
ALDH1A2	rs35853021	15	58388444	T>G	0.217	0.583	0.390	
ALDH1A2	rs2043085	15	58388755	C>T	0.457	0.133	0.866	
ALDH1A2	rs1532085	15	58391167	G>A	0.454	0.133	0.736	
ALDH1A2	rs148141131	15	58391234	C>G	0.057	0.048	0.211	
ALDH1A2	rs117497152	15	58395391	G>C	0.072	0.073	0.934	
ALDH1A2	rs493258	15	58395681	T>C	0.241	0.704	0.243	

ALDH1A2	rs920915	15	58396268	G>C	0.205	0.569	0.201	
ALDH1A2	rs16940212	15	58401821	G>T	0.288	0.366	0.140	
ALDH1A2	rs62000869	15	58414712	G>A	0.484	0.642	0.024	0.791
ALDH1A2	rs11857380	15	58420004	T>C	0.141	0.847	0.596	
ALDH1A2	rs117579778	15	58421680	G>T	0.278	0.816	0.076	
ALDH1A2	rs375372	15	58424416	T>G	0.226	0.894	0.923	
ALDH1A2	rs28665687	15	58425033	C>T	0.306	1.000	0.064	
ALDH1A2	rs16940262	15	58425229	T>C	0.468	0.225	0.955	
ALDH1A2	rs1373657	15	58425563	G>T	0.209	0.672	0.492	
ALDH1A2	rs148730463	15	58426100	A>C	0.106	0.005	0.152	
ALDH1A2	rs1077835	15	58431227	A>G	0.381	0.162	0.740	
ALDH1A2	rs1077834	15	58431280	T>C	0.373	0.273	0.899	
ALDH1A2	rs1800588	15	58431476	C>T	0.345	0.536	0.933	
ALDH1A2	rs2070895	15	58431740	G>A	0.372	0.425	0.964	
ALDH1A2	rs6494005	15	58432325	A>G	0.232	0.794	0.383	
ALDH1A2	rs8034802	15	58432593	A>T	0.345	1.000	0.334	
ALDH1A2	rs261334	15	58434545	C>G	0.321	0.200	0.648	
ALDH1A2	rs261332	15	58435126	G>A	0.097	0.600	0.224	
ALDH1A2	rs588136	15	58438299	T>C	0.349	0.756	0.954	
ALDH1A2	rs17301739	15	58438440	C>G	0.112	0.057	0.645	
ALDH1A2	rs261342	15	58438954	G>C	0.420	0.012	0.322	
ALDH1A2	rs485538	15	58448978	T>C	0.426	0.506	0.631	
ALDH1A2	rs75828665	15	58452490	A>G	0.053	1.000	0.822	
ALDH1A2	rs11071383	15	58452771	C>T	0.314	0.517	0.744	
ALDH1A2	rs182007	15	58453199	G>A	0.122	0.519	0.526	

ALDH1A2	rs3825776	15	58454631	C>T	0.484	0.780	0.511
ALDH1A2	rs12148704	15	58466207	G>A	0.372	0.690	0.923
ALDH1A2	rs7182229	15	58472984	G>T	0.121	0.659	0.458
ALDH1A2	rs34888457	15	58480024	C>T	0.441	0.925	0.796
ALDH1A2	rs34959352	15	58485127	T>C	0.109	0.336	0.125
ALDH1A2	rs35771822	15	58485408	A>C	0.088	0.380	0.528
ALDH1A3	rs9944290	15	100884530	A>G	0.410	0.386	0.234
ALDH1A3	rs56063524	15	100899614	A>C	0.122	1.000	0.717
ALDH1A3	rs4646690	15	100914804	G>C	0.059	0.003	0.701
ALDH1A3	rs1130738	15	100915635	A>G	0.268	0.812	0.816
ALDH1A3	rs1802603	15	100916579	G>A	0.058	0.002	0.573
ALDH1B1	rs4646771	9	38394809	C>T	0.385	0.844	0.964
ALDH1L1	rs142956509	3	126113796	G>T	0.058	1.000	0.281
ALDH1L1	rs118025802	3	126129267	C>T	0.088	0.071	0.727
ALDH1L1	rs2305225	3	126131662	A>G	0.439	0.570	0.979
ALDH1L1	rs2276724	3	126135566	T>C	0.189	0.166	0.684
ALDH1L1	rs4646712	3	126153139	G>A	0.335	0.060	0.541
ALDH1L1	rs4646703	3	126158019	C>T	0.118	1.000	0.290
ALDH1L1	rs1868138	3	126158188	T>A	0.288	0.909	0.614
ALDH1L1	rs80272064	3	126160389	A>T	0.054	1.000	0.147
ALDH1L1	rs9825571	3	126181318	A>G	0.442	0.850	0.486
ALDH1L1	rs1107366	3	126185322	G>A	0.499	0.217	0.096
ALDH1L1	rs10934753	3	126187336	G>A	0.288	0.650	0.053
ALDH1L1	rs79012325	3	126188313	T>C	0.050	1.000	0.906
ALDH1L1	rs1992857	3	126191161	T>G	0.364	0.689	0.424

ALDH1L2	rs12579790	12	105025202	C>T	0.166	0.736	0.214	
ALDH1L2	rs4964314	12	105037430	G>A	0.287	0.068	0.409	
ALDH1L2	rs12297353	12	105040522	G>A	0.188	0.363	0.272	
ALDH1L2	rs7297999	12	105042706	T>C	0.468	0.262	0.924	
ALDH1L2	rs11112345	12	105050162	G>A	0.067	0.711	0.922	
ALDH1L2	rs10861332	12	105056229	A>G	0.426	0.154	0.275	
ALDH1L2	rs73393842	12	105058419	G>A	0.060	0.671	0.040	0.306
ALDH1L2	rs10861337	12	105058664	G>A	0.249	0.079	0.226	
ALDH1L2	rs10861342	12	105065019	T>C	0.241	1.000	0.332	
ALDH1L2	rs2440693	12	105096145	C>T	0.424	0.504	0.332	
ALDH1L2	rs12830078	12	105099625	C>T	0.228	0.508	0.954	

Abbreviations: SNP: single nucleotide polymorphism; MAF: minor alleles frequency; HWE: Hardy-Weinberg equilibrium.

Table S3. Regulatory annotation of *ALDH1A2* rs16939929

Position	SNP ID	LD (r^2)	Reference allele	Alternate allele	ASN frequency	Enhancer histone marks	DNAse	Motifs changed	eQTL hits
58204734	rs16939929	1	A	G	0.11	LNG, FAT, MUS, SKIN		Hsf, PU.1, SPIB, p300	1 hit
58206020	rs74793808	0.95	G	A	0.12	FAT, SKIN, BLD	CRVX		
